# Leveraging cover crop functional traits and nitrogen synchronization for yield-optimized waxy corn production systems

**DOI:** 10.3389/fpls.2025.1591506

**Published:** 2025-06-03

**Authors:** Mengjing Sun, Long Zhang, Jiangkuo Zhou, Ziping Liu, Cong Peng, Zechen Jia, Yanjie Lv, Yongjun Wang

**Affiliations:** ^1^ College of Agronomy, Jilin Agricultural University, Changchun, Jilin, China; ^2^ Key Laboratory of Crops Physiological Ecology and Agronomy in Northeast China, Ministry of Agriculture of the People's Republic of China (MOA); Institute of Agricultural Resource and Environment, Jilin Academy of Agricultural Sciences, Changchun, Jilin, China

**Keywords:** waxy corn, cover crop, yield, nitrogen use efficiency, path analysis

## Abstract

**Context:**

Prolonged monoculture of waxy corn (*Zea mays* L. var. *ceratina Kulesh*) exacerbates soil nutrient depletion and compromises soil structural integrity, concomitant with underutilization of photosynthetically active radiation (PAR) resources.

**Objective:**

Implementing cover cropping post-harvest of waxy corn can utilize residual environmental resources for soil quality improvement. Nevertheless, the agronomic consequences of this practice on canopy architecture optimization and resource allocation dynamics in subsequent growing seasons require systematic elucidation.

**Methods:**

The regionally adapted cultivar Wannuo 2000 (W67×W68), predominantly cultivated in northeastern China, was employed for canopy characterization. Three experimental treatments were established, including waxy corn continuous monoculture control (CK), shamrock (*Trifolium pratense* L.) rotation cropping after waxy corn harvest (ZT) and rapeseed (*Brassica napus* L.) rotation cropping after waxy corn harvest (ZB). Each treatment incorporated five nitrogen (N) application gradients (0, 60, 120, 180, 240 kg N ha^−^¹) arranged in randomized complete block design (RCBD) with triplicate plots.

**Results:**

Cover crops integration significantly enhanced waxy corn productivity. Mean yields for ZT and ZB systems during 2022–2023 demonstrated 20.74% (8.88 ± 2.50 Mg ha^−1^) and 22.26% (8.99 ± 3.12 Mg ha^−1^) increases respectively compared to CK. Remarkably, under 25% N reduction scenarios, ZT and ZB achieved 15.25% (44.58 ± 6.28%) and 20.67% (46.68 ± 7.15%) improvements in nitrogen use efficiency (NUE) relative to conventional practice. The path analysis revealed synergistic interactions between cover crop incorporation and N management mediated through canopy structural optimization. Specifically, enhanced leaf area index (4.56 ± 0.69 m² m^−^²) and elevated pre-silking canopy N content (132.61 ± 26.33 g N plant^−1^) collectively drove post-silking biomass accumulation (134.88 ± 26.85 g plant^−1^) and N remobilization efficiency.

**Conclusion:**

The integrated cover crop-nitrogen reduction system enhanced both yield and NUE relative to monoculture benchmarks, demonstrating dual benefits in environmental conservation and agricultural productivity enhancement.

**Implication:**

This study establishes a theoretical framework and provides empirical evidence for the sustainable intensification of waxy corn production systems.

## 1 Introduction

In developing regions where food security remains precarious, reconciling agricultural productivity with ecological sustainability constitutes a pressing scientific challenge (Sanchez et al., 2020; Najafi Vafa et al., 2022). As a dual-purpose crop serving both dietary and economic functions, waxy corn (*Zea mays* L. var. *ceratina Kulesh*) occupies critical positions in food systems, though its yield intensification typically demands escalated agrochemical inputs and labor investments (Feng et al., 2024). The concomitant environmental externalities of nutrient oversupply, particularly nitrogen (N) leaching and phosphorous runoff, progressively compromise soil health parameters and aquatic ecosystem integrity, ultimately diminishing the economic viability and agroecosystem resilience of waxy corn cultivation (Motasim et al., 2024). This dichotomy necessitates innovative cultivation strategies that reconcile yield enhancement with ecological preservation in waxy corn production systems.

Proper N management exerts dual effects on pedospheric systems, demonstrating the ability to enhance soil nutrient availability and stable carbon pool formation. However, excessive application accelerates SOC mineralization, reducing carbon sequestration potential while compromising yield stability (Chen et al., 2009). The environmental ramifications extend beyond terrestrial ecosystems, as surplus N undergoes biogeochemical transformations into nitrous oxide (N_2_O), a potent greenhouse gas contributing to climate change (Winnick, 2021; Cui et al., 2024). Sustainable agricultural intensification requires three fundamental approaches: 1) Regional optimization of N application thresholds through precision agricultural techniques, 2) Development of crop cultivars with enhanced N assimilation efficiency, and 3) Implementation of integrated nutrient management systems (Wang et al., 2020; Duan et al., 2024a). Recent investigations further highlight the critical role of legacy N from optimized fertilization regimes in sustaining subsequent crop N demand, thereby improving systemic nitrogen use efficiency (Zhao et al., 2023).

The Soil Science Society of America formally defines cover crops as non-commodity vegetation strategically implemented to maintain continuous soil coverage during agricultural fallow periods. These biological systems provide multidimensional agroecological benefits through canopy closure, organic matter deposition, and rhizosphere engineering, with leguminous species exhibiting particular efficacy in atmospheric N assimilation via symbiotic rhizobia associations (Hubbard et al., 2013). Beyond N enrichment, cover crop systems confer critical ecosystem services including erosion mitigation through aggregate stabilization, hydrological regulation via improved infiltration capacity, pathogen suppression through allelochemical production, and carbon sequestration in stabilized organic fractions (Seitz et al., 2024). The integrated effects of enhanced microbial biodiversity and optimized soil physicochemical properties collectively contribute to agricultural system resilience. Current evidence substantiates that incorporating cover cropping aligns with global sustainability frameworks by addressing soil conservation, climate change mitigation, and yield stability imperatives (Purwanto and Alam, 2019).

Solar radiation constitutes a primary biophysical driver governing plant developmental process, with direct regulatory effects on photosynthetic efficiency, canopy water-use dynamics, and biomass partitioning mechanisms (Durand et al., 2021; Wang et al., 2022). In maize cultivation systems, optimized canopy architecture development enhances photosynthetically active radiation (PAR) interception, subsequently driving carbohydrate assimilation and yield formation (Soto-Gómez and Pérez-Rodríguez, 2022). The practice of maize-legume intercropping demonstrates particular efficacy in this context, where symbiotic interactions stimulate maize population establishment while maintaining radiation capture efficiency. However, suboptimal light environments may induce photomorphogenic adaptations affecting leaf angle distribution and stomatal patterning, necessitating strategic canopy management to balance light harvesting with metabolic expenditure (Yang et al., 2021). Contemporary agronomic research consequently positions canopy structure optimization as a cornerstone strategy for yield intensification, particularly through manipulation of leaf area index (LAI) and vertical foliage arrangement to maximize grain yield under fluctuating radiation regimes (Croce et al., 2024).

Waxy corn production systems confront dual agronomic constraints: suboptimal yield potential and inefficient N utilization, issues that concurrently compromise economic viability and exacerbate environmental externalities (Lu et al., 2024; Elrys et al., 2023). Addressing this sustainability paradox requires innovative cultivation strategies that synergistically enhance productivity while optimizing N economy—a critical nexus for ensuring both food security and ecological stewardship. The post-harvest phase in northern China’s waxy corn systems presents untapped potential, with residual photosynthetic active radiation and accumulated thermal units providing agronomic windows for cover crop integration. This investigation systematically elucidates the mechanistic relationships between cover cropping practices and subsequent crop performance through a comprehensive analysis of N remobilization dynamics and photo assimilate partitioning patterns, ultimately establishing an evidence base for sustainable intensification pathways.

## 2 Materials and methods

### 2.1 Experimental location description

**Figure 1:**
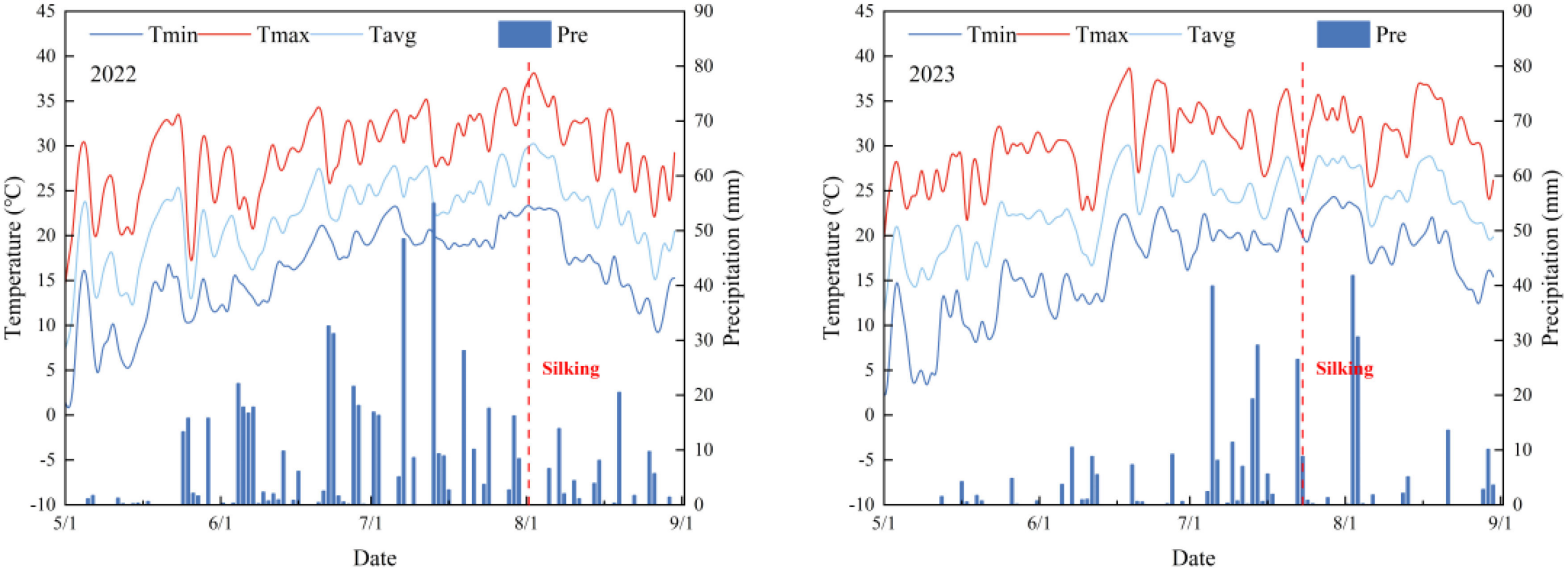
The mean daily maximum temperature (Tmax), mean daily minimum temperature (Tmin), mean average temperature (Tavg), precipitation (Pre) during waxy corn growing season in 2022 and 2023.

The two-year field experiment (2022-2023) was conducted at Gongzhuling Experimental Station (43°52’N, 124°81’E; 206 m asl) within the Northeast China Corn Belt, characterized by a temperate continental monsoon climate with 2,744.32 annual sunshine hours and 4,262.71 MJ m^−^² solar radiation. Daily air temperature and precipitation during the experimental years are presented in **Figure 1**. Significant interannual precipitation variation was observed, with growing-season totals measuring 598.20 mm in 2022 and 339.80 mm (−43.20%) in 2023, inducing moderate drought conditions. Before the trial initiation, the soil contained 25.22 g kg^−1^ of organic matter, 78.67 mg kg^−1^ of alkali N, 197.67 mg kg^−1^ of available K, and 22.18 mg kg^−1^ of available P. Comparative soil analysis (0–20 cm depth) between monoculture (CK) and cover crop-integrated systems (ZT&ZB) revealed progressive soil quality enhancement: organic matter content increased by 5.00% (2021) and 9.76% (2022), accompanied by respective 5.88%/18.06%, 8.20%/19.75%, and 16.54%/28.00% improvements in alkaline hydrolysable nitrogen, available potassium, and phosphorus concentrations (**Table 1**). These findings demonstrate the temporal accumulation effect of cover crops on soil fertility parameters.

**Table 1:** Chemical properties of test site soil at 0–20 cm depth. IR, the average increase ratio of ZT and ZB relative to CK.

Year	Treatment	pH	Organic matter (g kg^−1^)	Alkali N (mg kg^−1^)	Available K (mg kg^−1^)	Available P (mg kg^−1^)
2022	CK	7.41	22.93	79.33	150.33	24.69
	ZT	7.48	24.65	84.00	168.67	29.24
	ZB	7.45	23.50	84.00	156.67	28.30
	IR	0.81%	5.02%	5.88%	8.20%	16.54%
2023	CK	7.32	23.50	84.00	147.67	26.29
	ZT	7.50	26.37	102.67	178.33	39.54
	ZB	7.58	25.22	95.67	175.33	27.76
	IR	3.01%	9.76%	18.06%	19.75%	28.00%

### 2.2 Experimental design and agronomic management

This study is a long-term positioned experiment initiated in 2021, therefore, the location of the plots for each treatment has remained unchanged between different years. In the preceding years, the field had been under continuous grain maize cultivation with full-season fertilizer applications of 210 kg N ha^−^¹, 90 kg P ha^−^¹, and 100 kg K ha^−^¹. The study employed a split-plot randomized complete block design with triplicate replications, incorporating three agronomic variables: planting systems (CK: post-harvest fallow; ZT: *Trifolium pratense* L. rotation cropping; ZB: *Brassica napus* L. rotation cropping) and N application gradients (0, 60, 120, 180, 240 kg N ha^−^¹). Fifteen treatment combinations were systematically arranged in 38.4 m^2^ experimental plots (5 m × 7.8 m) with 1.5 m isolation buffers, utilizing the regionally adapted waxy corn cultivar ‘Wannuo 2000’ (W67×W68) predominant in Northeast China’s agricultural production systems.

**Figure 2:**
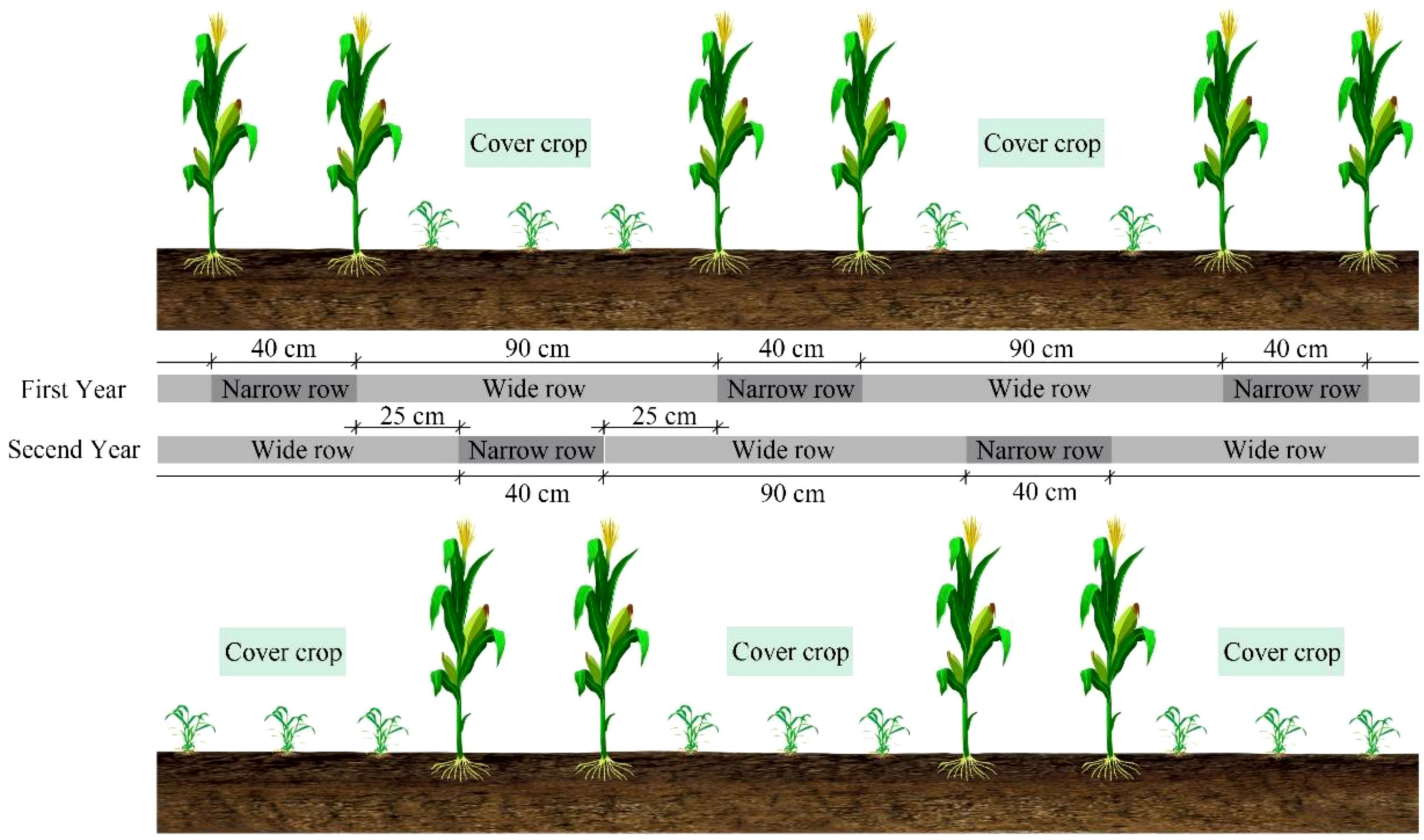
Schematic diagram of planting methods for corn and cover crops.

The agronomic protocol implemented a rotational cover crop integration system, cover crops had been planted after the waxy corn harvest in the previous year. Waxy corn was cultivated under wide-narrow row configuration (0.9 m/0.4 m row spacing) during 5 May - 19 August 2022 and 27 April - 17 August 2023 growing windows, as planted at a density of 70,000 plants ha^–1^. The cover crops were sown on August 20, 2022, and August 18, 2023, with shamrock planted at a seeding rate of 110 kg ha^−^¹ and rapeseed (cultivar Longyou 7) at a planting density of 330,000 plants ha^−^¹. Before planting waxy corn, a rotary tiller was used to incorporate the cover crops into the soil to a depth of 15 cm, and then plant the waxy corn above the incorporated cover crop. After harvesting the waxy corn, cover crops were planted in wide rows between the waxy corn stubble to avoid root interference (**Figure 2**). Nutrient management comprised basal application (V3 stage) of 90 kg P_2_O_5_ ha^−^¹, 90 kg K_2_O ha^−^¹, and 50% total N allocation, with the remaining N top-dressed at the V12 stage through precision banding. Hydrological inputs relied exclusively on precipitation events, complemented by integrated pest management strategies to maintain phytosanitary conditions.

### 2.3 Sampling and measurements

Yield quantification was conducted at the fresh food period (R3) through destructive sampling of central plot areas (3 m × 3 row section). A representative sampling protocol was implemented, selecting five ears per plot for multivariate analysis: 1000-kernel mass (g), kernel count per ear, ear density (ears ha^−^¹), and grain yield (Mg ha^−^¹ at 14% moisture content).

Plant biomass dynamics were assessed through phased destructive sampling at critical phenological stages: silking (R1) and fresh food period (R3). Triplicate plant specimens per plot were subjected to desiccation (85°C forced-air oven, 48-hour duration) until mass stabilization (± 0.01 g precision). The homogenized tissue samples (≤ 0.50 mm particle size) underwent N quantification via micro-Kjeldahl digestion (CN61 M/KDY-9820, Beijing, China) following AOAC 978.02 protocols. Derived parameters included:


Post-silking N uptake amount=R3 N content–R1 N content

Post-silking N uptake percentage in total N=Post-silking N uptake amount/R3 N content

N remobilization=R1 vegetative organ N content–R3 vegetative organ N content

Nitrogen use efficiency (NUE)=(R3 N content in N fertilized cropland–R3 N content in N unfertilized cropland)/Total N input to cropland

Post-silking dry matter (DM) weight=R3 DM–R1 DM

Harvest index (HI)=Grain dry weight/Aboveground biomass

Canopy radiation dynamics were quantified during the critical silking phase (R1 stage) in both experimental years using a SunScan canopy analysis system (Delta-T Devices Ltd., Cambridge, UK). High-resolution photosynthetically active radiation (PAR) measurements were conducted between 10:00-12:00 solar time under clear-sky conditions (cloud cover< 20%), capturing incident radiation above the canopy and transmitted PAR (TPAR) at four critical canopy strata: ground level, third leaf below ear node, ear node position, and third leaf above ear node. Intercepted PAR (IPAR) was derived through radiative transfer modeling of these vertical attenuation profiles.

Concurrent leaf area characterization employed a non-destructive sampling protocol, measuring length-width dimensions (with 0.75 correction factor) on five representative plants per plot. Leaf area index (LAI) was calculated as the ratio of total foliar surface area (m² plant^−^¹) to projected ground occupancy (m² plant^−^¹). The canopy light extinction coefficient (*K*) was determined through Beer-Lambert law application:


$$K=-\frac{1}{\text{LAI}}\text{ln}\left(\frac{{\text{PAR}}_{\text{transmiteed}}}{{\text{PAR}}_{\text{incident}}}\right)$$

Where PAR_transmiteed_ is the IPAR of waxy corn canopy, and PAR_incident_ is the TPAR.

### 2.4 Statistical analysis

Statistical analyses were conducted by using Origin 2022 (OriginLab, Northampton, Massachusetts, USA). The data sources were analyzed through a three-way ANOVA followed by the LSD test to compare the differences among the groups. Pearson correlation coefficients were calculated using SPSS to identify interrelationships among the measured parameters, and path correlation analyses were conducted to better understand causal relationships.

## 3 Results

### 3.1 Grain yield and yield components

Agronomic interventions significantly influenced waxy corn productivity through both main and interactive effects (*p*< 0.01), as detailed in **Table 2**. The brassica-based rotation cropping system (ZB) under N_180_ fertilization demonstrated peak yields of 12.18 Mg ha^−^¹ (2022) and 11.96 Mg ha^−^¹ (2023), surpassing conventional monoculture (CK) by 22.26% across both seasons. Yield component analysis revealed systemic improvements in ZT and ZB systems: 16.02% higher ear density, 2.71% elevated kernel count per ear, and 14.44% increased 1000-kernel mass relative to CK.

**Table 2:** Yield and yield components of different treatments under different N conditions in 2022 and 2023.

Year	Nitrogen (kg ha^−1^)	Treatment	Harvest ear per ha	Kernel number per ear	1000-kernel weight (g)	Grain yield (Mg ha^−1^)
2022	N_0_	CK	16524 a	482 c	263.12 b	2.73 b
		ZT	26781 a	502 a	294.94 a	4.33 a
		ZB	25071 a	498 b	302.67 a	2.92 b
	N_60_	CK	41026 a	564 a	295.88 b	6.36 b
		ZT	43875 a	531 b	308.63 a	9.12 a
		ZB	50143 a	567 a	304.44 a	8.47 a
	N_120_	CK	55271 b	566 b	303.59 c	8.93 b
		ZT	68376 a	569 b	359.55 a	10.53 a
		ZB	61539 a	573 a	320.48 b	10.24 a
	N_180_	CK	51852 b	569 b	307.29 b	8.74 c
		ZT	54131 ab	579 a	334.13 a	11.04 b
		ZB	58690 a	584 a	347.74 a	12.18 a
	N_240_	CK	56411 a	572 a	319.51 a	10.07 a
		ZT	57550 a	575 a	313.95 a	10.46 a
		ZB	59829 a	578 a	335.81 a	10.86 a
2023	N_0_	CK	30200 a	424 c	274.72 c	1.72 b
		ZT	39316 a	439 a	339.16 a	4.07 a
		ZB	34188 a	434 b	320.20 b	3.45 a
	N_60_	CK	52422 b	482 c	292.60 b	8.22 b
		ZT	58120 ab	552 a	340.34 a	9.31 a
		ZB	68376 a	527 b	333.84 a	8.93 a
	N_120_	CK	54701 b	543 c	285.11 c	8.78 b
		ZT	65527 a	566 a	359.55 b	10.94 a
		ZB	61539 ab	554 b	377.89 a	10.54 a
	N_180_	CK	51852 a	541 b	323.60 c	8.90 c
		ZT	60399 a	564 a	407.40 b	10.49 b
		ZB	60399 a	564 a	418.98 a	11.96 a
	N_240_	CK	57550 a	552 a	335.70 c	9.10 b
		ZT	59829 a	561 a	350.93 b	8.52 b
		ZB	71795 a	560 a	398.15 a	10.37 a
Year			***	***	***	ns
Nitrogen			***	***	***	***
Treatment			***	***	***	***
Year × Nitrogen			***	***	***	***
Year × Treatment			ns	***	***	ns
Nitrogen × Treatment			ns	***	***	**
Year × Nitrogen × Treatment			ns	***	***	***

Within each year, different lowercase letters of the same N in the same column showed the significant difference between each datum at *p*< 0.05. ***Significant at *p* ≤ 0.001; ns, non‐significant (LSD).

**Figure 3:**
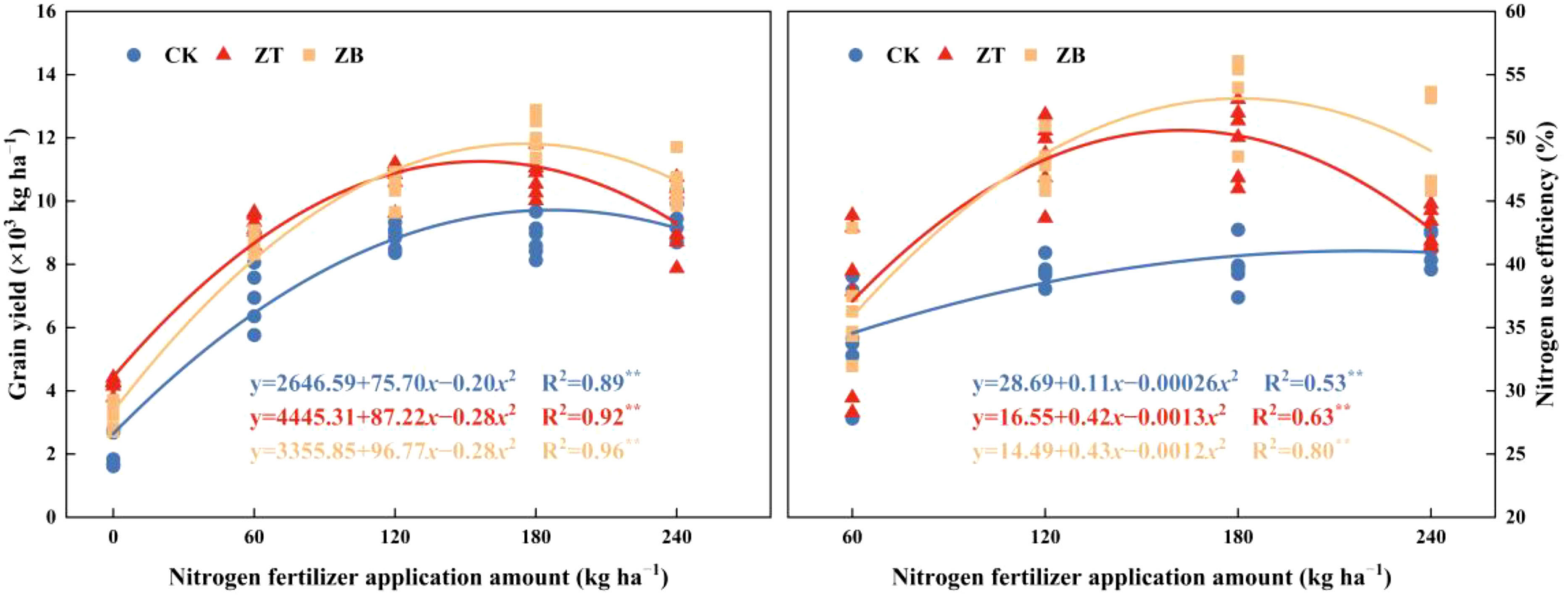
The impact of N fertilizer application amount on grain yield and nitrogen use efficiency (NUE) under different cover crop treatments. **Significant at *p* ≤ 0.01.

N response curves at CK showed yield increases slowly with N escalation (34.72 kg yield gain per kg N in 2022; 31.73 kg in 2023). Maximum productivity in ZT and ZB occurred at N_180_, with marginal yield gains of 49.12, 53.83 kg per kg N (2022) and 40.22, 61.68 kg (2023) respectively. Quadratic plateau modeling identified optimal N thresholds of 189.25 kg ha^−^¹ for CK, 155.75 kg for ZT, and 172.80 kg for ZB (**Figure 3**), demonstrating cover crops’ nitrogen-sparing capacity.

### 3.2 N accumulation, post-silking N uptake, and N remobilization

N dynamics analysis revealed significant treatment effects (*p*< 0.01) on pre-silking N allocation, post-silking assimilation efficiency, and system-level N utilization (**Table 3**). Both N application and cover crop integration demonstrated interactive effects across measured parameters. Maximum nitrogen use efficiency (NUE) was achieved at N_240_ for monoculture (CK: 41.24%), while cover crop systems (ZT: 49.89%; ZB: 54.28%) peaked at reduced N levels (N_180_), representing 15.25% and 20.67% efficiency improvements over CK respectively.

**Table 3:** N uptake and remobilization under different treatments in 2022 and 2023.

Year	Nitrogen (kg ha^−1^)	Treatment	N content at silking (g plant^−1^)	N content at R3 (g plant^−1^)	Post-silking N uptake (g)		Total N remobilization (g plant^−1^)	Nitrogen use efficiency (%)
					Amount (g plant^−1^)	Percentage in total N (%)		
2022	N_0_	CK	0.73 c	1.08 c	0.35 b	0.32 b	0.05 b	–
		ZT	0.93 a	1.56 a	0.63 a	0.40 a	0.12 a	–
		ZB	0.82 b	1.44 b	0.62 a	0.43 a	0.13 a	–
	N_60_	CK	1.03 c	1.38 c	0.35 b	0.25 b	0.18 a	35.30 a
		ZT	1.21 a	1.85 a	0.64 a	0.35 a	0.17 a	33.88 a
		ZB	1.11 b	1.73 b	0.62 a	0.36 a	0.21 a	34.29 a
	N_120_	CK	1.37 b	1.76 c	0.39 c	0.22 b	0.26 a	39.39 b
		ZT	1.52 a	2.36 a	0.84 a	0.36 a	0.13 b	46.44 a
		ZB	1.50 a	2.24 b	0.74 b	0.33 a	0.16 b	46.74 a
	N_180_	CK	1.50 b	2.10 b	0.60 b	0.29 b	0.24 a	39.68 c
		ZT	1.86 a	2.78 a	0.92 a	0.33 a	0.12 b	47.63 b
		ZB	1.81 a	2.79 a	0.98 a	0.35 a	0.13 b	52.64 a
	N_240_	CK	1.64 b	2.48 b	0.84 b	0.34 a	0.19 b	40.65 b
		ZT	2.05 a	2.98 a	0.93 a	0.31 a	0.19 b	41.55 b
		ZB	2.06 a	3.01 a	0.95 a	0.32 a	0.27 a	46.05 a
2023	N_0_	CK	0.40 c	0.72 c	0.32 b	0.44 b	0.05 b	–
		ZT	0.71 a	1.32 a	0.61 a	0.46 ab	0.12 a	–
		ZB	0.60 b	1.15 b	0.55 a	0.48 a	0.07 b	–
	N_60_	CK	0.55 c	1.01 c	0.46 b	0.46 a	0.04 c	33.23 a
		ZT	0.99 a	1.66 a	0.67 a	0.40 b	0.24 a	40.09 a
		ZB	0.82 b	1.47 b	0.65 a	0.44 ab	0.16 b	38.23 a
	N_120_	CK	0.86 c	1.40 c	0.54 b	0.39 c	0.13 b	39.47 b
		ZT	1.12 a	2.19 a	1.07 a	0.49 b	0.21 a	50.78 a
		ZB	0.89 b	1.98 b	1.09 a	0.55 a	0.11 b	48.46 a
	N_180_	CK	1.04 c	1.75 b	0.71 b	0.41 a	0.16 c	39.93 c
		ZT	1.52 a	2.66 a	1.14 a	0.43 a	0.27 a	52.14 b
		ZB	1.49 b	2.58 a	1.09 a	0.42 a	0.20 b	55.91 a
	N_240_	CK	1.26 c	2.15 b	0.89 b	0.41 a	0.22c	41.82 b
		ZT	1.59 b	2.83 a	1.24 a	0.44 a	0.29 b	44.16 b
		ZB	1.71 a	2.90 a	1.19 a	0.41 a	0.36 a	51.13 a
Year			***	***	***	***	ns	***
Nitrogen			***	***	***	***	***	***
Treatment			***	***	***	***	***	***
Year × Nitrogen			***	ns	***	***	***	ns
Year × Treatment			***	***	ns	***	***	*
Nitrogen × Treatment			***	***	***	***	***	***
Year × Nitrogen × Treatment			***	ns	*	***	***	ns

Within each year, different lowercase letters of the same N in the same column showed the significant difference between each datum at *p<* 0.05. *Significant at *p* ≤ 0.05; ***Significant at *p* ≤ 0.001; ns, non‐significant (LSD).

Cover crop treatments enhanced N acquisition capacity throughout the growth cycle, with ZT and ZB systems exhibiting 30.06-40.18% greater plant N content at silking (R1) and 23.41-34.49% elevation at fresh food period (R3) compared to CK. Post-silking N assimilation proved particularly responsive to cover crop integration, showing 54.55-61.99% increases in absolute uptake (kg N ha^−^¹), and 5.21-26.06% increases proportional contribution to total N under N60-N180 regimes. Notably, this advantage diminished at excessive N levels (N_240_), where soil mineral N saturation likely negated cover crops’ rhizosphere modification benefits.

Mechanistically, Correlation analysis identified post-silking N assimilation (correlation coefficient 0.76) and R3 N status (0.67) as primary NUE determinants (**Table 4**). The N amplification effect (ΔNUE/N input) for cover crop systems exceeded CK by 6.13-10.40%, demonstrating superior N conversion efficiency through improved soil-plant N synchrony.

**Table 4:** Correlation analysis between N content accumulation or N uptake percentage on total N and yield or yield composition in waxy corn.

Index	N content at silking	Post-silking N uptake	N content at R3	Total N remobilization	N uptake percentage in total N
Grain yield	0.73 ^**^	0.67 ^**^	0.77 ^**^	0.50 ^**^	−0.13 ^ns^
Harvest ear per ha	0.54 ^**^	0.61 ^**^	0.62 ^**^	0.53 ^**^	0.03 ^ns^
Kernel number per ear	0.80 ^**^	0.56 ^**^	0.78 ^**^	0.61 ^**^	−0.42 ^ns^
1000-kernel weight	0.43 ^**^	0.82 ^**^	0.63 ^**^	0.48 ^**^	0.37 ^**^
NUE	0.48 ^**^	0.76 ^**^	0.67 ^**^	0.20 ^ns^	0.30 ^*^

*Significant at *p* ≤ 0.05; **Significant at *p* ≤ 0.01; ns, non‐significant (LSD).

### 3.3 Dry matter accumulation

Interannual biomass partitioning patterns demonstrated significant three-way interactions among growing seasons, N regimes, and cover crop systems (*p<* 0.01, **Table 5**). The 43.20% precipitation deficit in 2023 intensified drought stress, amplifying cover crops’ biomass enhancement effects compared to the optimal 2022 conditions. Across both experimental years, cover crop integration systematically improved dry matter (DM) accumulation across growth phases and harvest index (HI), exhibiting N-dependent efficacy gradients. Notably, ZT outperformed ZB under low-moderate N inputs (N_0_-N_120_), while this relationship reversed under high N regimes (N_180_-N_240_), suggesting N-mediated interspecific competition dynamics.

**Table 5:** Dry matter production (DM) and harvest index (HI) under different cover crop treatments and various N fertilizers from 2022 and 2023, respectively.

Year	Nitrogen (kg ha^−1^)	Treatment	Pre-silking DM (g plant^−1^)	R3 DM (g plant^−1^)	Post-silking DM (g plant^−1^)	Harvest index
2022	N_0_	CK	70.51 c	114.43 b	43.93 c	0.19 c
		ZT	90.21 a	144.45 a	54.24 b	0.29 a
		ZB	80.86 b	140.52 a	59.66 a	0.25 b
	N_60_	CK	101.50 c	148.20 c	46.69 c	0.19 c
		ZT	118.08 a	186.11 a	68.03 a	0.23 a
		ZB	113.29 b	176.88 b	63.59 b	0.22 b
	N_120_	CK	116.35 d	162.00 c	45.65 c	0.20 b
		ZT	135.96 a	234.17 a	98.21 a	0.24 a
		ZB	128.21 b	204.92 b	76.72 b	0.20 b
	N_180_	CK	128.46 c	204.37 c	75.91 c	0.22 b
		ZT	147.91 b	241.29 b	93.38 b	0.23 a
		ZB	157.51 a	280.08 a	122.58 a	0.22 b
	N_240_	CK	131.09 c	226.06 b	94.97 a	0.23 c
		ZT	133.49 b	213.47 c	79.98 b	0.27 a
		ZB	150.05 a	250.14 a	100.09 a	0.25 b
2023	N_0_	CK	64.87 c	98.32 b	33.45 b	0.27 b
		ZT	89.63 a	145.80 a	56.17 a	0.32 a
		ZB	75.31 b	138.08 a	62.77 a	0.31 a
	N_60_	CK	77.58 c	127.80 c	50.22 b	0.24 b
		ZT	109.53 a	183.91 a	74.38 a	0.36 a
		ZB	100.69 b	163.96 b	63.27 ab	0.34 a
	N_120_	CK	95.92 c	154.37 c	58.45 c	0.27 b
		ZT	122.83 a	215.98 a	93.14 b	0.34 a
		ZB	109.25 b	209.10 b	99.86 a	0.34 a
	N_180_	CK	99.32 c	159.07 c	59.75 c	0.28 b
		ZT	133.54 b	231.18 b	97.64 b	0.34 a
		ZB	138.08 a	252.34 a	114.26 a	0.32 a
	N_240_	CK	111.75 c	170.95 c	59.20 b	0.30 b
		ZT	117.42 b	187.86 b	70.43 b	0.33 a
		ZB	127.12 a	215.34 a	88.22 a	0.34 a
Year		***	***	***	**	***
Nitrogen		***	***	***	***	***
Treatment		***	***	***	***	***
Year × Nitrogen		***	***	***	***	***
Year × Treatment		***	***	***	***	***
Nitrogen × Treatment		***	***	***	***	***
Year × Nitrogen × Treatment		***	***	***	***	***

Within each year, different lowercase letters of the same N in the same column showed the significant difference between each datum at p< 0.05. **Significant at p ≤ 0.01, ***Significant at p ≤ 0.001 (LSD).

Quantitative analysis revealed, in 2022, the average pre-silking DM of ZT and ZB were 14.19% and 14.97% higher than CK; the average post-silking DM were 28.22% and 37.60% higher than CK; meanwhile, the HI increased 22.33% and 10.68% than CK, respectively. In 2023, the average pre-silking DM of ZT and ZB were 27.48% and 22.47% higher than CK; the average post-silking DM were 50.05% and 64.08% higher than CK; meanwhile, the HI increased 24.26% and 21.32% than CK. Maximum whole-plant biomass (280.08 g plant^−^¹ in 2022; 252.34 g plant^−^¹ in 2023) occurred in ZB under N_180_, mirroring yield optimization patterns. N responsiveness followed quadratic trends, peaking at N_180_ before declining, indicative of metabolic sink limitations under excessive fertilization.

Biometric correlations established strong positive associations between cumulative DM and yield parameters (r = 0.57-0.87, *p*< 0.01), whereas HI showed no significant relationship (r = 0.11, *p* > 0.05), confirming biomass accumulation rather than partitioning efficiency as the primary yield determinant in these systems (**Table 6**). The differential nitrogen × cover crop × environment interactions highlight the necessity for adaptive management strategies balancing N inputs with cover crop functional types under variable precipitation regimes.

**Table 6:** Correlation analysis between dry matter (DM) accumulation or harvest index (HI) and yield or yield composition in waxy corn. .

Index	Pre-silking DM	Post-silking DM	R3 DM	HI
Grain yield	0.87 ^**^	0.76 ^**^	0.85 ^**^	0.11 ^ns^
Harvest ear per ha	0.67 ^**^	0.58 ^**^	0.65 ^**^	0.30 ^*^
Kernel number per ear	0.86 ^**^	0.62 ^**^	0.78 ^**^	− 0.12 ^ns^
1000-kernel weight	0.57 ^**^	0.74 ^**^	0.68 ^**^	0.62 ^**^

*Significant at *p* ≤ 0.05; **Significant at *p* ≤ 0.01; ns, non‐significant (LSD).

### 3.4 Silking and maturity leaf area index and extinction coefficient

**Figure 4:**
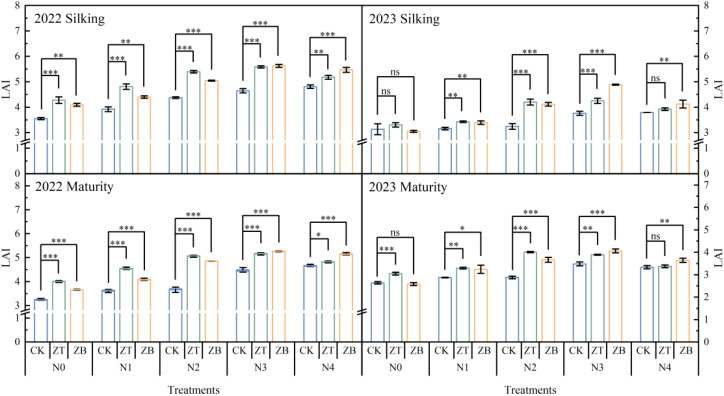
LAIs of waxy corn under different N fertilizer and cover crop treatments at silking and maturity in 2022 and 2023. *Significant at *p* ≤ 0.05; **Significant at *p* ≤ 0.01; ***Significant at *p* ≤ 0.001; ns, non‐significant (LSD).

Canopy architectural analysis revealed systematic improvements in leaf area index (LAI) under cover crop integration, with ZT and ZB systems exhibiting 17.81 and 17.76% superior LAI enhancement compared to monoculture (CK) across critical growth stages (**Figure 4**). In the optimal 2022 season, maximum LAI values of 5.58 (ZT) and 5.62 (ZB) at silking under N180 fertilization significantly outperformed CK by 18.54% and 15.66% respectively, demonstrating N-mediated canopy expansion. This advantage persisted through maturity, with ZT and ZB maintaining 17.08 and 19.89% greater LAI, indicative of prolonged photosynthetic capacity.

**Figure 5:**
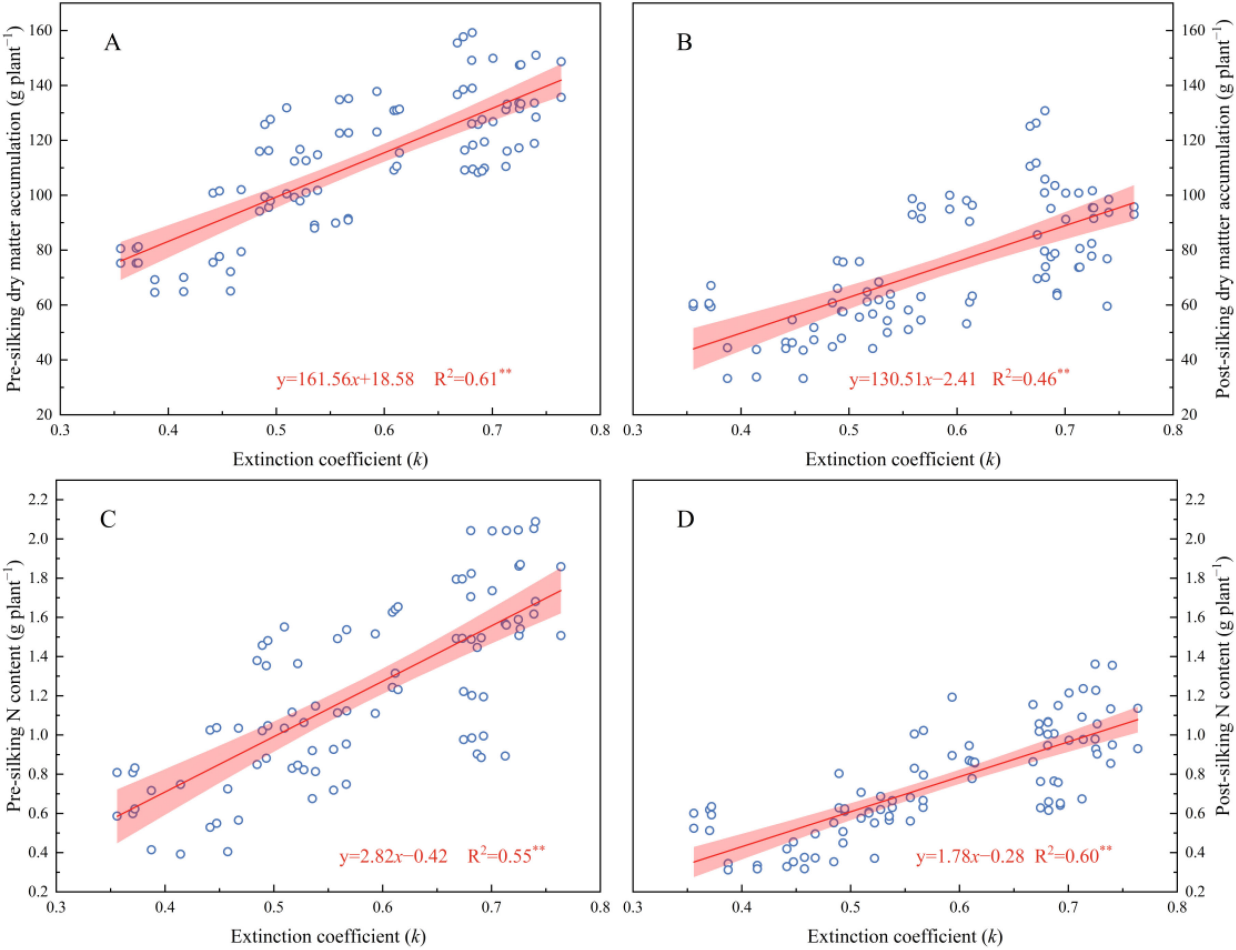
The correlation between extinction coefficient (*k*) and pre-silking DM **(A)**, post-silking DM **(B)**, pre-silking N content **(C)**, and post-silking N content **(D)**, respectively.

The drought-affected 2023 season modified these relationships - while low (N_0_) and excessive (N_240_) N inputs negated LAI differences between systems, moderate fertilization (N_60_-N_180_) sustained ZT/ZB’s canopy superiority (15.59-15.89% over CK). Mechanistically, LAI dynamics directly regulated light extinction coefficients (k) through Beer-Lambert law interactions, where k increased logarithmically with LAI escalation. This light attenuation pattern drove biomass accumulation, evidenced by strong k correlations with pre-silking (R²=0.61) and post-silking (R²=0.46) dry matter production (**Figure 5**). The synergistic LAI-k relationships underscore cover crops’ dual role in optimizing both light interception efficiency and N utilization precision, particularly under 180 kg N ha^−^¹ fertilization where canopy architecture and radiation use efficiency reached equilibrium.

### 3.5 Path analysis

**Figure 6:**
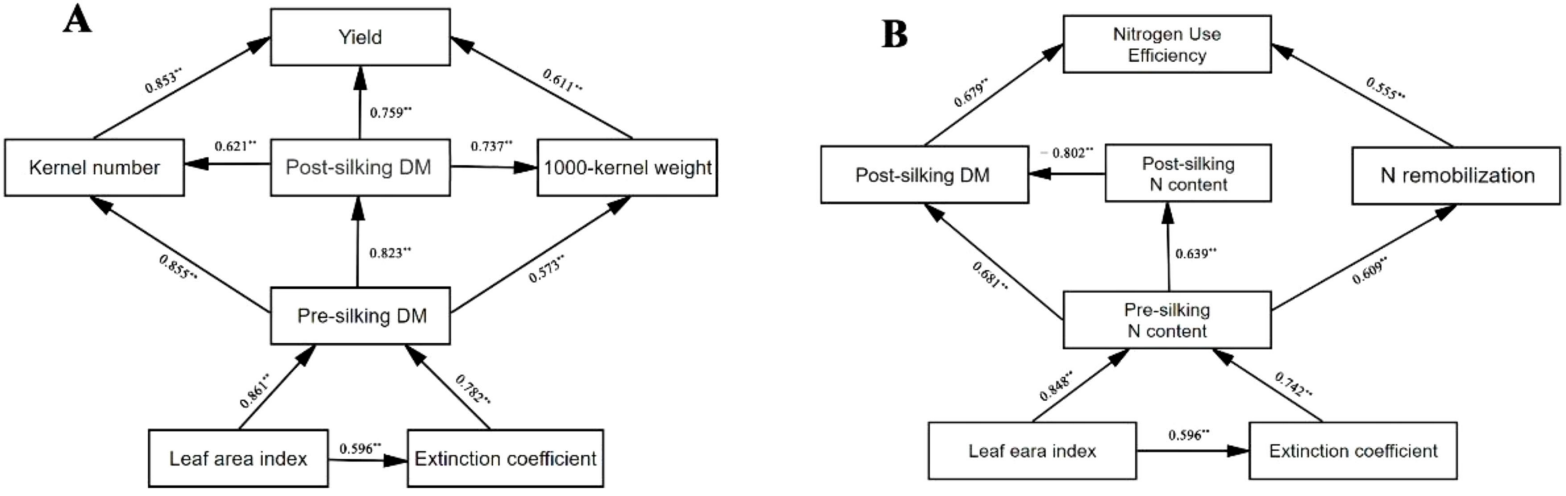
Path analysis of the relationship among yield, kernel number, 1000-kernel weight, pre-silking dry matter (DM), post-silking DM, leaf area index and extinction coefficient **(A)**, and the relationship among nitrogen use efficiency, post-silking DM, pre-silking N content, post-silking N content, N remobilization, leaf area index and extinction coefficient **(B)**. Arrows point from causes and effects, and solid lines represent direct effects. The values along of the arrows are r of the correlation with ** being *p* ≤ 0.01.

Factors affecting the yield and NUE, and the relationship between these causes and effects, are shown in **Figure 6**. The path analysis revealed that leaf area index (LAI) and extinction coefficient (*k*) exerted significant direct effects on pre-silking dry matter (DM), with standardized path coefficients (β) of 0.92 and 0.39, respectively. Pre-silking DM subsequently demonstrated substantial direct effects on three yield components: kernel number (β = 0.86), post-silking DM (β = 0.96), and 1000-kernel weight (β = 0.57). These yield components directly influenced final grain yield through distinct pathways, with kernel number showing the strongest association (β = 0.46), followed by post-silking DM (β = 0.42) and 1000-kernel weight (β = 0.19). Regarding nitrogen dynamics, LAI and *k* significantly affected pre-silking N content through direct pathways (β = 0.63 and 0.37, respectively). Pre-silking N content subsequently influenced three critical nitrogen-related parameters: post-silking DM (β = 0.29), post-silking N content (β = 0.64), and N remobilization (β = 0.61). The NUE was primarily determined by post-silking nitrogen content (β = 0.55) and nitrogen remobilization (β = 0.38), demonstrating their differential contributions to nitrogen utilization.

The results showed further evidence that the interplay of cover crop and N significantly influenced yield and NUE by canopy structure. Canopy architectural improvements mediated by cover crop integration fundamentally enhanced radiation capture efficiency and N utilization dynamics across growth stages. The synergistic interaction between cover crop systems and N management amplified leaf area development, with cover crop treatments consistently outperforming monoculture in both optimal and drought-affected seasons. Enhanced leaf area indices prolonged photosynthetic activity duration while optimizing light distribution patterns within the canopy, driving coordinated improvements in pre-silking resource accumulation and post-silking translocation efficiency.

## 4 Discussion

Contemporary agroecological research substantiates cover crops as multifunctional biological regulators within agricultural ecosystems, demonstrating tripartite benefits through soil nutrient modulation, erosion mitigation, and systemic soil health enhancement (Sharma et al., 2017; Tribouillois et al., 2022; Goettl et al., 2024). These living mulches operate through three synergistic mechanisms: 1) temporal niche complementarity in nutrient acquisition, 2) rhizosphere engineering via root exudate-mediated microbial consortia, and 3) physical armor against erosional forces through canopy-litter composite layers. Particularly in maize production systems, cover crops exhibit dual-phase optimization—enhancing vegetative growth through microclimate moderation while concurrently implementing nutrient sequestration mechanisms that reduce fertilizer dependency (Sainju et al., 2005; Muñoz et al., 2014). The experimental outcomes substantiate the resilience-enhancing capacity of optimized cover crop systems under climatic variability. Across contrasting growing seasons, cover crop integration with precision N management demonstrated sustained yield superiority over conventional monoculture, particularly under drought-stressed conditions (**Table 2**). While interannual precipitation fluctuations induced significant reductions in biomass accumulation and N assimilation, the ZB system maintained robust productivity—exhibiting 34.00-39.00% yield advantage under optimal N application (N_180_) compared to control plots. This drought-mitigation efficacy underscores the system’s ability to buffer environmental stressors through enhanced soil moisture retention and N cycling efficiency.

### 4.1 Effects of cover crops and N application on NUE in waxy corn

The differential performance observed between normal and stress years highlights a critical synergy between cover crop functional traits and N availability to stabilize yield outcomes under hydrological uncertainty. The observed yield enhancement mechanisms align with established principles of ecological intensification, where cover crops functionally compensate for soil N limitation through species-specific strategies. Under N-deficient regimes (N_0_-N_120_), the leguminous cover crop (*Trifolium pratense* L.) likely demonstrated superior efficacy through symbiotic N fixation, effectively bypassing soil N constraints and elevating plant-available N pools during critical growth phases. This biological N provisioning likely translated directly into enhanced kernel initiation and grain filling efficiency. Conversely, brassica cover crops (*Brassica napus* L.) exhibited maximum performance synergies under optimized N inputs (N_180_-N_240_), where their extensive biomass production likely stimulated microbial-mediated N mineralization and improved edaphic conditions through enhanced aggregate stability. The N-dependent functional complementarity between cover crop types underscores the importance of matching species selection with fertilization regimes to maximize system-level productivity. These findings corroborate global evidence of cover crops’ capacity to mitigate nutrient limitations while amplifying yield components through both direct and indirect pathways (Gabriel and Quemada, 2011; Radicetti et al., 2016). Mechanistic analysis revealed a N-dependent yield optimization threshold governed by Liebig’s law of the minimum, where yield and NUE exhibited quadratic response patterns to fertilization intensity (**Figure 3**). This relationship delineates an economic-environmental equilibrium threshold—the precise N input level that simultaneously maximizes productivity and minimizes agrochemical surplus. Beyond this critical threshold (N_180_ in cover crop systems), N saturation likely induced metabolic constraints through luxury consumption, triggering assimilates partitioning imbalances that preferentially favored vegetative over reproductive development. The establishment of such N-mediated yield ceilings underscores the imperative for precision N management protocols that harmonize crop physiological demands with ecological carrying capacities, achieving dual optimization of productivity and sustainability in waxy corn production systems.

Strategic N management through agroecological intensification has emerged as a pivotal approach for reconciling agricultural productivity with environmental stewardship (Abdo et al., 2024; Zou et al., 2024). Cover crops function as dynamic biofilters within this paradigm, mediating soil N cycles through three synergistic mechanisms: rhizosphere immobilization of residual N, microbial reactivation of legacy nutrients, and physical-chemical stabilization of soil organic matter. By establishing continuous biological activity during fallow periods, these systems synchronize N mineralization pulses with subsequent crop demand while suppressing nitrification-denitrification cascades that drive greenhouse gas emissions (Baptistella et al., 2020; Hirsh et al., 2021). The resultant enhancement of N residence time and plant availability fundamentally reconfigures traditional input-output relationships, enabling yield stability under reduced fertilization regimes. This ecological engineering strategy exemplifies the transition from linear nutrient management to circular nutrient economies in modern agroecosystems. This study demonstrates that cover crop integration significantly improves NUE across fertilization regimes, with synergistic nitrogen × cover crop interactions driving non-linear response patterns (**Table 3**). The temporal alignment between enhanced N accumulation during silking—a critical phase for grain differentiation and yield formation (Jacobs and Pearson, 1992)—and cover crop-mediated N provisioning highlights a fundamental physiological linkage. This N assimilation advantage during early reproductive development establishes a cascade effect, optimizing subsequent nutrient partitioning efficiency while minimizing environmental losses through improved N demand-supply synchrony. The diminished NUE enhancement at extreme N levels (N_60_-N_240_) underscores the importance of balanced fertilization strategies that harmonize agroecological intensification with crop developmental phenology. During critical reproductive phases, the developing grain sink exerts a hierarchical pull-on N resource (Zhang et al., 2016), amplifying translocation efficiency from vegetative tissues—a process modulated by cover crop functional traits. Under moderate N regimes (N_60_-N_180_), leguminous and brassica systems demonstrated superior N channeling capacity compared to monoculture, reflecting improved source-sink coordination through enhanced rhizosphere N availability. This synergy diminished under excessive N inputs (N_240_), where soil N saturation likely shifted N cycling toward organic matter stabilization pathways rather than immediate plant accessibility. The N flux redirection suggests a threshold-dependent transition in cover crop functionality: from direct N provisioning under limited conditions to long-term soil N reservoir building in high-input scenarios. The drought-affected 2023 season revealed a paradoxical N assimilation pattern: despite marked reductions in vegetative-stage (R1-R3) N content across treatments, post-silking N acquisition and allocation efficiency exceeded 2022 levels, particularly in cover crop systems. This counterintuitive phenomenon stems from drought-induced rhizosphere modification, where early-season water deficits triggered adaptive root architectural remodeling—characterized by enhanced antioxidant enzyme activity and lignification—that improved hydraulic conductivity and nutrient foraging capacity (Wu et al., 2024).

### 4.2 Effects of cover crops and N application on dry matter accumulation in waxy corn

Drought-adaptive dry matter partitioning mechanisms in waxy corn were fundamentally reconfigured through cover crop integration, demonstrating a compensatory strategy that prioritizes reproductive sink strength under hydrological constraints. While vegetative biomass accumulation (R1-R3) decreased during the drought-affected season, the system exhibited enhanced assimilate remobilization efficiency and harvest index elevation—a paradoxical response indicating metabolic prioritization of grain filling over vegetative persistence (**Table 5**). This drought-induced source-sink realignment was amplified in cover crop systems, where ZT and ZB treatments increased post-silking photo assimilate allocation rates compared to monoculture, suggesting improved phloem loading capacity and sucrose synthase activity during critical grain formation phases citation. These findings substantiate cover crops’ role as biostimulants of plant resource optimization, effectively decoupling yield formation from environmental stochasticity through improved assimilate partitioning precision. N management analysis revealed distinct dry matter accumulation patterns between production systems (Zhu et al., 2021). The monoculture control (CK) exhibited a linear increase in biomass accumulation with escalating N inputs, while cover crop systems demonstrated quadratic responses, peaking at N_180_ fertilization (**Table 5**). This N optimization threshold corresponded with maximum yield realization, indicating synchronized source-sink coordination. Notably, supra-optimal N application (N_240_) induced metabolic sink limitations in cover crop systems, reducing translocation efficiency from vegetative tissues to developing grains. The results substantiate that cover crop integration necessitates N input calibration, with 180 kg N ha^−^¹ establishing the equilibrium point between ecological intensification and agronomic productivity in waxy corn systems (**Figure 3**).

### 4.3 Effects of cover crops on canopy structure and light interception capacity in waxy corn

Leaf Area Index (LAI) serves as a critical biophysical determinant of canopy light interception efficiency and photosynthetic optimization in cereal cropping systems (Liu et al., 2022; Duan et al., 2024b). This study demonstrates that cover crop integration fundamentally reconfigures canopy architectural dynamics in subsequent waxy corn, with functional divergence between leguminous (*Trifolium pratense* L.) and brassica (*Brassica napus* L.) systems across N gradients (**Figure 4**). The leguminous cover crop exhibited superior LAI enhancement under N-limited regimes (N_0_-N_120_), leveraging symbiotic N fixation to sustain canopy expansion. Conversely, brassica dominance emerged under high N availability (N_180_-N_240_), where its extensive biomass production synergized with soil N mineralization to optimize light distribution patterns (Zhang et al., 2020). These N-mediated canopy modifications establish a self-reinforcing cycle—LAI augmentation facilitates radiation-use efficiency improvement, which in turn sustains photosynthetic capacity during reproductive transitions (Rens et al., 2018). The observed species × N interaction underscores the importance of matching cover crop functional types with fertilization strategies to maximize light capture efficiency while maintaining source-sink balance throughout crop developmental phases (**Figure 5**). Under N-limited conditions, legume-derived N provisioning sustains canopy expansion, while brassica dominance under high-N regimes promotes multi-layered light distribution through biomass-mediated architectural modifications. Path analysis confirms LAI’s pivotal role as a biophysical lever, simultaneously amplifying both carbon assimilation and N utilization precision through optimized source-sink dynamics (**Figure 6**). The findings establish canopy architectural modulation as a central strategy for decoupling productivity from environmental constraints in sustainable maize production systems.

This study establishes a novel ecological intensification framework for waxy corn production through functional integration of cover crop-mediated canopy optimization and N synchronization strategies. The demonstrated hierarchical mechanism—spanning from rhizosphere N provisioning to photosynthetic efficiency amplification—reveals how strategic species × fertilization pairings reconfigure source-sink coordination, achieving unprecedented yield-NUE synergies. Crucially, the systems approach resolves the historical dichotomy between input reduction and productivity enhancement by engineering self-sustaining light-nutrient coupling effects. These conceptual advances provide a blueprint for sustainable intensification, where managed biodiversity replaces chemical inputs through ecological leverage points in canopy architecture and nutrient cycling. The findings pioneer a paradigm shift from conventional yield-centric management to whole-system resource optimization, positioning cover crop integration as a keystone strategy for decoupling cereal production from environmental externalities while maintaining economic viability.

## 5 Conclusion

Cover crops (shamrock and rapeseed) integration significantly enhanced waxy corn productivity, and enables sustainable intensification through light-nutrient synergy and resource optimization. This approach reduces N demand while enhancing yield stability via improved canopy-mediated radiation capture and drought-adaptive N remobilization. The demonstrated coordination of ecological processes—soil nutrient stewardship, hydrological buffering, and carbon-N coupling—provides a scalable template for climate-resilient agriculture. By transforming post-harvest periods into productive ecological phases, the system redefines conventional monoculture paradigms, achieving simultaneous productivity gains and environmental stewardship through managed biodiversity.

## Data Availability

The raw data supporting the conclusions of this article will be made available by the authors, without undue reservation.
